# Medical Education and Research in India: A Teacher’s Perspective

**DOI:** 10.7759/cureus.24680

**Published:** 2022-05-02

**Authors:** Venkataramana Kandi

**Affiliations:** 1 Clinical Microbiology, Prathima Institute of Medical Sciences, Karimnagar, IND

**Keywords:** medical students, medical education technology, india, national medical commission, competency, physician, research, medical education

## Abstract

Medical education is a systematic process wherein interested and eligible individuals are trained to become physicians/surgeons. It is assumed that a person who completes the Bachelor of Science and Bachelor of Medicine (MBBS) degree will be competent enough to perform the duties of a physician of first contact. However, it is not the case with graduates from India. Most MBBS graduates prefer to pursue a postgraduate degree and become unavailable to people or governments. The doctor-to-patient ratio in India (1:1,655) does not currently satisfy the World Health Organization’s prescribed ratio (1:1,000). The Government of India, therefore, has been taking initiatives to increase the number of MBBS graduates. Moreover, there are several doubts over the quality of medical education and the competency of medical students. In addition, the National Medical Commission, the epic body that regulates the medical education and practice in India, has recently been conducting medical education technology workshops to improve teachers and has devised a new curriculum to elevate the standards of medical education in India. This editorial attempts to provide readers with the current status of medical education and research in India.

## Editorial

The Indian medical education system has recently seen a makeover in the form of a change in the curriculum. This change may have become inevitable due to growing concerns, both within the country and globally, over the low standards and quality of MBBS graduates. It has been widely accepted that medical students graduating from Indian institutions are not competent enough to practice. This is evident by the requirement for medical graduates from India to clear the United States Medical Licensing Examination (USMLE) to practice in the United States. Therefore, the National Medical Commission (NMC), previously known as the Medical Council of India (MCI), has come forward with a vision to revamp medical education in India. The main motto of this initiative by the NMC is to ensure that Indian medical graduates (IMG) are competent enough to function as community physicians of first contact [1]. This decision by the Government of India (GOI) was taken after considering the high disease burden among people, as well as the huge disparities in the status of medical institutions/establishments/facilities/services within different geographical regions of the country. Moreover, the NMC has made it mandatory for all medical teachers to undergo a training program on medical education technology (MET). The NMC regularly conducts basic and advanced workshops/courses for all medical teachers in the country. However, the NMC appears to have ignored the most significant aspect that could potentially contribute to the success of a teacher/learner in medical education. The attitudes of both the teachers and learners significantly influence the learning outcomes, thereby affecting the competency of medical graduates. Carefully designed faculty development programs may contribute to medical teachers’ professionalism, management, and leadership abilities that further enable students to become competent physicians [2].

The current scenario

Most medical teachers, especially those who participate in the teaching of students in their I MBBS and II MBBS may be considered accidental teachers. Although this is not usually considered a topic for discussion, it must be noted that the first two years of the MBBS course function as a foundation for students. During this period, students learn basic subjects, including anatomy, physiology, biochemistry, pathology, microbiology, and pharmacology. Basic and comprehensive knowledge of these subjects is considered extremely useful to understand the clinical subjects that the students pursue from the third year of the MBBS course. However, in practice, disinterested teachers are misguiding students to learn the subjects from an examination point of view rather than emphasizing the importance of the role played by pre and para-clinical subjects for the rest of their future in the medical profession (clinical relevance and integrated approach). The scenario may be the same with teachers who opt for less familiar clinical degrees after having compromised on their specialization of interest. Unfortunately, specializations gain familiarity based on the expected earnings they can get during the practice and not particularly about the interest/skills they have concerning the subject/specialization.

The NMC and GOI’s decision to improve the doctor-to-patient ratio has indirectly affected the teacher-to-student ratio. With increased numbers of medical institutions being permitted and more in the pipeline, there is a continuous movement of faculty resulting in transient deficiencies of faculty that directly affects the quality of curriculum delivery despite the improvement/change in the curriculum, as envisaged by the NMC recently. Further, the NMC and the GOI must consider the numbers of MBBS and MD (Doctor of Medicine) graduates who move out of the country for livelihood and other reasons. Interestingly, not many medical graduates were available who volunteered during the initial days of the coronavirus disease 2019 (COVID-19) pandemic. Therefore, public and private healthcare facilities were forced to work with medical students (house surgeons/interns/final MBBS students) and fresh MBBS graduates. The main reason is that MD graduates belonging to the pre and para-clinical subjects, sparing a few, lose their connectivity to medicine and lack the confidence to examine patients. This aspect needs serious consideration by the GOI, NMC, local governments, as well as the doctors themselves, who should make efforts to provide their services by running evening community clinics.

Admission for entry into the MBBS course is currently carried out based on reservations following the caste system that is prevalent in India. A significant percentage of seats are filled based on the student’s capability to pay fees, and some are accommodated to the non-resident Indian (NRI) category. Similarly, the admission of students is based on a competitive multiple-choice-based examination that does not evaluate students’ interests and attitudes and merely evaluates their theoretical knowledge. These are the main reasons for the deteriorating quality of medical graduates in India. Conversely, the deterioration in quality comes from the fact that the concepts that were described in the medical education workshops are not pursued by the majority of faculty members across medical colleges in India. Compared to the olden days, currently, teachers are not spending enough time with students and patients, especially in the clinical departments.

Medical teachers

Teachers appointed in medical institutes are required to have an MD degree in the respective subjects. However, the NMC has allowed the appointment of non-medical teachers (teachers without an MBBS degree) for up to 30% (50% in biochemistry) in both pre and para-clinical subjects excluding the department of pathology [3]. This was mainly due to the lack of qualified MD teachers available for the job. Recently, the change in the medical education curriculum with more emphasis on competency and competency-based medical education (CBME) has forced the NMC to reconsider the appointment of non-medical teachers. Therefore, NMC has decided to reduce the number of non-medical teachers in the anatomy, physiology, and biochemistry departments, and stop the recruitment of such teachers to the microbiology and pharmacology departments. The decision of the NMC regarding non-medical teachers was long pending with complaints about such teachers emerging over the years both from students as well as teachers with MD degrees. The major reason for the NMC’s decision to minimize and remove non-medical teachers is because the GOI and the NMC have already given a green signal/approval for admission into several MD courses in these departments. On the contrary, many MBBS graduates who fail to pursue a clinical MD/MS degree have been opting for non-clinical (pre and para-clinical) MD subject specialization courses. However, this decision by the NMC had some drawbacks. The major one is the lack of interest among MBBS graduates admitted in the concerned subject of specialization. After joining the courses, these students face difficulties in completing the course at the designated time. Moreover, the mandatory dissertation required for them to complete the course is not done with the required/desired technical and ethical standards.

In contrast to a Ph.D. degree, wherein the pursuant does research work for at least three years to become eligible for the award of the degree, MD graduates work as part-time teachers (tutor/junior resident/senior resident) and are able to complete the course within three years. All universities in India come under the aegis of the Union Grants Commission (UGC). However, medical institutions and medical universities function under the umbrella of the NMC. The UGC mandates that Ph.D. students publish at least two original research articles in reputed journals and present at least one abstract in a national/international conference to become eligible to be awarded the degree. This is in complete contrast to the MD degree, wherein the students only undertake research work for a maximum period of one year, and it is not mandatory to publish/present papers in journals/conferences. Moreover, the period during the MD study is considered a teaching experience in the position of resident/tutor/demonstrator. Therefore, MD degree holders become eligible for appointments as assistant professors after the study period. This points to the fact that an MD degree must not necessarily be considered a research degree. The UGC has a mechanism wherein the Ph.D. thesis and postgraduate dissertations submitted by the students are deposited in a repository (Shodhganga) and are carefully screened for plagiarism before they are approved/accepted. The Information and Library Network Centre (INFLIBNET) is an autonomous interuniversity center of the UGC [4]. However, even today, this practice is not followed by medical universities. Therefore, MD dissertations are generally not as standard as Ph.D. theses. Medical Universities, in the future, should consider screening all MD dissertation submissions for potential plagiarism and make the dissertations available in the public domain (similar repository as the INFLIBNET). Several institutions do not support the financial aspects of the MD dissertation, and this limitation could be the reason why the students are unable to pursue real research and finally end up writing dissertations without even working on the topic. This situation can be improved by active vigilance by the NMC and at the institutional level regarding the MD dissertations and their standards. Given the above observations, the matter of equivalence of an MD degree with a Ph.D. degree appears to be questionable. However, medical universities in India and autonomous research institutes funded by the GOI are accepting/recognizing MD degree holders as Ph.D. guides. Such practices should be re-evaluated by higher education bodies/councils because it affects the standards of Ph.D. holders and their future in research activities.

Medical research

Medical teachers who were not properly trained in research end up being not interested in pursuing research after obtaining their degrees. This probably was the main reason why the NMC was particular about the research publications requirement for promotions. However, medical teachers find it difficult to do even these mandatory research publications. This is majorly attributed to the lack of financial and logistic support from the public and private administrations. The lack of proper research orientation among medical teachers appears to have significantly impacted the research inclination of students. Moreover, medical institutions have not been supportive of the research activities of both the faculty and students. The GOI, under the Indian Council for Medical Research, has been implementing limited numbers of short-term studentship (STS) research fellowships (two-month duration) for medical students [5]. Despite this initiative, many more interested students will remain unbenefited. This issue may be addressed by the institution by devising a mechanism wherein research-oriented faculty may be roped into a group (research wing) that mainly functions to facilitate interested students to pursue research work under able faculty members. Because research work involves financial implications, medical institutions must create a fund (crowdfunding, donations, corpus, others) that potentially serves this purpose.

The criteria for promotion that requires a medical teacher to have published at least two papers in an indexed journal also appear to be responsible for the deterioration of research standards among medical teachers and institutions. This decision by the NMC was instrumental in the emergence of several predatory journals that claim to have been indexed by the agencies recognized/prescribed by the NMC. However, the NMC realized and changed its decision and excluded the Index Copernicus as the desired indexing agency that was responsible for the emergence of pay-to-publish journals. Interestingly, the NMC recently added the Indian Citation Index to the list of desired indexing agencies along with others that are already present, including PubMed, Scopus, DOAJ, and EMBASE, among others. Given unethical research and publication practices, the NMC may choose to accept literature reviews as an acceptable type of article in consideration for promotions. This will enable faculty members to pursue research and get increasingly acquainted with current trends with extremely limited resources and encourage within-country and foreign collaborations.

Other concerns

**Figure 1:**
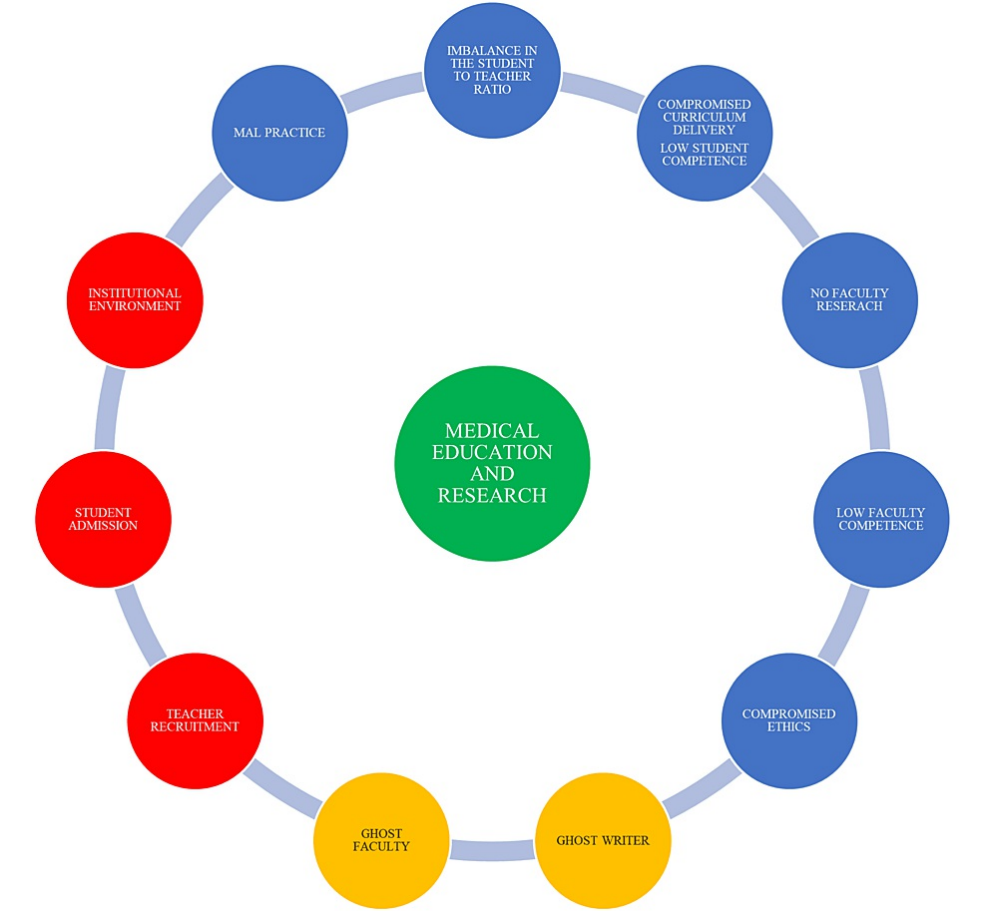
Pictorial representation of the factors that affect medical education and research.

Despite several positive changes by the NMC, medical institutions are further plagued by the problems of ghost faculty, ghostwriters, and other malpractices related to examinations, research, dissertation work, and publications. The factors that potentially affect medical education and research in a medical institution are depicted in Figure 1.

Ghostwriter

Ghostwriters are anonymous persons who write manuscripts (that in this case are represented by research publications) for teachers who require them for promotions. A ghostwritten research article is used by a teacher to his/her credit by implanting his/her name on it and without mentioning the person who has written it. Moreover, ghostwritten articles are not necessarily written based on authentic data collected after conducting bench work. These amount to malicious practices under the recommendations by the Committee of Publication Ethics (COPE). Ghostwriters have been in full function helping several medical teachers to acquire research publications required for promotions. The problem with ghostwriting is that authors claim a work neither by doing nor by writing. Such literature that is written without actually working and available as published content in the public domain will adversely influence the scientific community and public health.

Ghost faculty

It has been a frequent occurrence in the past decade, wherein medical teachers representing more than one medical college appear before the NMC during inspections. However, NMC did identify this problem and punished several such medical faculties. There was also a problem of duplicate faculty wherein the certificates of a medical person were used to wrongly represent the faculty. Despite strict vigilance by the NMC, the newest trend is the ghost faculty. Medical colleges call upon qualified medical graduates during the inspection and show them as resident faculty members to the NMC. It is an open secret that is followed ubiquitously by several institutions throughout India. Interestingly, the faculty list shown by the institutes could range between 15 and 20 in each department during the inspection days. However, once the NMC inspection is completed, the actual faculty that works on the ground is considerably low in numbers (hardly 10). This significantly affects the faculty-to-student ratio. Assuming a college has 200 students, and the college shows 15 staff during the inspection, the actual number of teachers working on the ground will be less than 10. This results in overcrowding during practical demonstrations, and, for this reason, many students do not follow/understand the practical aspects. Moreover, with a smaller number of faculty members, teachers do not function in the manner that allows them to train each student adequately. Several practicing doctors and academically uninterested graduates are approached by the institutions. Due to the financial favors offered by institutions, medical graduates agree to be on the rolls of the college.

The ghost faculty who regularly present themselves to the NMC during the inspection do not even take a single theory/practical class. However, institutes promote them regularly based on the number of years of experience, despite the fact that the person has never attended the college in person, nor has he/she taken a single class. The ghost faculty issue emerges because there is a frequent increase in the number of medical colleges.

Admission requirements for entry into the MBBS course also require serious considerations. More than half of the admissions currently are carried out based on reservations based on the caste system that is prevalent in India. Although the remaining seats are considered open, a significant percentage are filled based on the student’s capability to pay fees, and some are accommodated as NRIs. This implies that the merit of students is grossly ignored, and admissions, therefore, in the current situation do not guarantee the entry of qualified students who are willing and motivated to pursue the course.

Currently, India has the highest number (>600) of medical colleges in country-wise statistics. However, the medical education quality and standards of medical graduates have been under the scrutiny of the GOI and the NMC. A mere change in the curriculum and faculty training does not necessarily guarantee standards. Increased vigilance, uniform salaries, peer evaluation of research publications used for promotions, support and encouragement for faculty pursuing research, continuing medical education in the form of workshops/seminars/symposiums/conferences, and extensive student feedback are mandatory to further strengthen the quality of medical education and improve the standards of medical graduates in India. Recently, the UGC has decided to appoint non-Ph.D. teachers in universities for utilizing their vast experience and expertise. It is, therefore, imperative that subject expertise matters much more than the degree a person holds.

Teachers are role models for students, and to nurture future generations in the right direction, it is more than essential to improve the attitudes and quality of teachers. Further, it is the institutes that teach the society and the country as a whole regarding how systematically one should function to achieve desirable results. Therefore, institutes must take responsibility for establishing the best educational environment.

In conclusion, it should be noted that addressing the deteriorating standards and quality of medical education requires a multifaceted approach. The implementation of the new curriculum for undergraduates and medical education technology workshops for faculty members does not guarantee an improvement in the quality. Dedication from admitted students, faculty, and institution administration is equally important.
